# Diet and Psoriasis‐Related Information on Instagram: A Quality and Content Analysis of Posts Under Popular Psoriasis Hashtags

**DOI:** 10.1111/jhn.70034

**Published:** 2025-03-04

**Authors:** Sabrina Cowan, Poppy Hawkins, Ghislaine Marks, Rosalind Fallaize

**Affiliations:** ^1^ School of Life and Medical Sciences University of Hertfordshire Hatfield UK

**Keywords:** dermatology, diet, Instagram, nutrition, psoriasis, social media

## Abstract

**Background:**

Psoriasis is a chronic immune‐mediated skin condition. Evidence supporting dietary management of psoriasis is limited. However, People Living with Psoriasis (PLwP) trial dietary interventions as a management strategy, often taken from popular literature. Social media is a popular source of nutrition information. However, little is known about the dietary information suggested for psoriasis on these platforms. The aim of this study was to explore the dietary approaches suggested for psoriasis on Instagram and evaluate their quality.

**Methodology:**

Cross‐sectional content analysis of Instagram posts providing dietary information for plaque psoriasis management, posted under the 12 most popular psoriasis hashtags. Posts were evaluated for quality using the DISCERN instrument.

**Results:**

Overall, 138 Instagram posts were analysed. The most common type of dietary recommendation was ‘exclusion’, most frequently of alcohol and dairy. Detox and ‘clean eating’ were also frequently mentioned, as well as the inclusion of protein, and adherence to a gluten‐free diet. The most common content creators were those with those with lived experience of psoriasis (29.7%). None of the posts were created by qualified nutrition professionals and only 6.5% were by healthcare professionals (HCPs). The majority (99%) of posts identified were of ‘poor’ quality.

**Conclusion:**

Most dietary information on Instagram for managing psoriasis is poor quality, restrictive, unsubstantiated and shared by non‐HCPs. Therefore, PLwP may be subject to dietary misinformation when using Instagram. HCPs should be equipped to counter diet and psoriasis misinformation. Further research is needed to investigate appropriate ways to provide dietary support for PLwP.

## Summary

- Most of the psoriasis‐related dietary information on Instagram is of poor quality and few posts are created by healthcare professionals. None of the dietary information identified was shared or created by a qualified nutrition professional.
- The most common dietary approach recommended was exclusion, most commonly suggesting the exclusion of alcohol or dairy.
- The findings suggest that PLwP may be subject to low‐quality and unsubstantiated dietary recommendations, whether they search for this type of information or not.
- These insights suggest that it is crucial for healthcare professionals to provide dietary support to PLwP to combat misinformation and minimise harm from following unsubstantiated dietary recommendations seen on Instagram.

## 1 Introduction

Psoriasis is a chronic, immune‐mediated skin disease that affects an estimated 60 million people worldwide [1, 2]. It is theorised to occur due to a combination of genetic and environmental factors. Psoriasis is associated with a range of co‐morbidities, including cardiovascular disease and metabolic syndrome [3, 4] and can have a substantial impact on quality of life [3, 5, 6, 7]. There are several types of psoriasis; the most common type, accounting for approximately 80%–90% of all cases of psoriasis globally, is plaque psoriasis [2]. Plaque psoriasis typically presents as raised red or grey scaly plaques on the skin [2]. The term ‘psoriasis’ is used throughout this paper to refer to ‘plaque psoriasis’.

There is no cure for psoriasis, and treatment is focused on symptom management [2]. The evidence for the dietary treatment of psoriasis is limited [8]. The most robust evidence is for low‐calorie diets in people living with obesity or overweight [9], and studies also suggest that following a gluten‐free diet in those with coeliac disease or gluten sensitivity [8, 10] could improve psoriasis symptoms. Observational studies suggest that the Mediterranean diet may have a beneficial impact on psoriasis [11, 12, 13], yet more research is needed to confirm this. A healthy balanced diet and alcohol reduction is suggested to help PLwP manage the associated comorbidities; however, there are no comprehensive dietary guidelines for psoriasis [14]. Despite this, evidence suggests that People Living with Psoriasis (PLwP) commonly try dietary interventions yet rarely discuss these dietary changes with a healthcare professional (HCP) [15].

Research indicates that in the absence of dietary guidelines, PLwPs are self‐prescribing dietary modifications suggested in the popular literature [14]. These are often restrictive [15] and could have detrimental effects on health and well‐being if followed without the guidance of an HCP [16, 17]. Sourcing dietary information online is becoming increasingly prevalent, particularly on social media platforms [18, 19]. Instagram is the 5th largest social media platform globally, with 1 billion monthly active users [20, 21]. It has rapidly developed into a platform where both personal and professional content is shared [20, 22]. Instagram allows any content creator, defined as an individual or entity who generates and publishes content including photos, videos and infographics on the platform, to share health information, regardless of their qualification or sources of information [18, 23, 24, 25]. Research has suggested that Instagram is commonly used by PLwP to find information about psoriasis [26]. Emerging evidence suggests that content relating to psoriasis treatments on social media platforms such as Instagram [24], Twitter [27, 28], YouTube [29, 30] and TikTok [31] is of poor quality, unsubstantiated by evidence, and rarely generated by HCPs [24, 28, 31, 32, 33]. However, no studies have specifically explored the content, quality and creators of dietary‐specific information for psoriasis available on Instagram.

Recognising the large role that social media now plays in the dissemination of health information, this study aims to explore the type of dietary information recommended, the content creators sharing this information, and evaluate the quality of dietary information posted on Instagram for the management of plaque psoriasis in adults. Exploring the previously unexamined dietary recommendations promoted on social media for psoriasis will offer unique insight for qualified nutrition professionals, dermatologists and other HCPs involved in psoriasis care. Enabling them to be better equipped to provide comprehensive, patient‐centred dietary support to PLwP.

## 2 Methods

This was a cross‐sectional study that explored the dietary approaches suggested for psoriasis posted on Instagram under popular psoriasis hashtags and evaluated the quality of each post using the DISCERN instrument [34]. The DISCERN instrument is a validated tool designed to enable users to evaluate the quality of health information [34]. The findings are reported according to the Strengthening the Reporting of Observational Studies in Epidemiology (STROBE) guidelines [35].

### 2.1 Search Strategy and Data Collection

**Figure 1:**
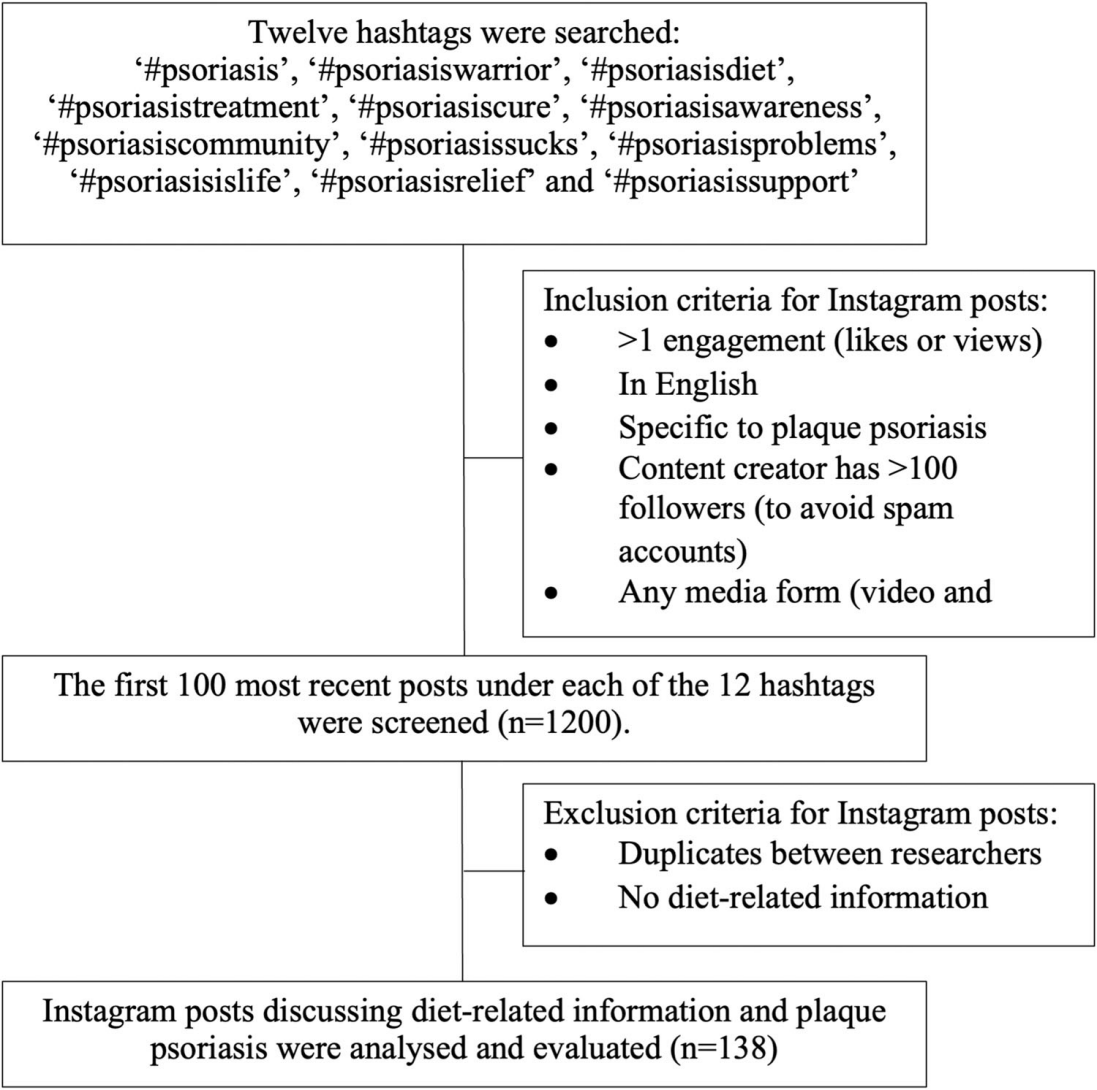
Study design flow chart. Twelve hashtags were searched on Instagram to collect public data on the management of plaque psoriasis regarding the type of dietary information and content creator.

Data were collected by searching for pre‐defined social media hashtags on Instagram for information about diet and psoriasis. To identify these hashtags, a preliminary search was conducted using ‘#psoriasis’ as a prefix and noting psoriasis‐related hashtags containing the largest number of posts, to ensure that only the most popular hashtags were included; a benchmark of 10,000 posts was deemed sufficient to achieve this, in line with previous studies [24, 29]. Twelve hashtags identified met this criterion, see Figure 1. At the time of searching, between 10,000 and 1.9 million posts were listed under each hashtag. Data collection occurred between 11th and 14th December 2022.

Posts listed under these hashtags were filtered by ‘most recent’ using a feature on the Instagram platform and the first 100 posts under each hashtag were collected. This minimised the effect of Instagram algorithms biasing the results and is a methodology used by similar research [24]. The inclusion criteria required posts to have been created in English in either picture or video form, specific to plaque psoriasis, by a content creator with > 100 ‘followers’ (to avoid spam accounts) and must have been engaged with at least once (via likes or views). Hashtags were searched and collected by two researchers (S.C. and G.M.).

### 2.2 Data Categorisation and Analysis

The Instagram posts were categorised depending on the type of dietary approach. The categories were not pre‐established but were decided during the analyses guided by the content of the posts. Some of the Instagram posts contained more than one category of dietary approach. These were split and counted as separate dietary approaches for descriptive analysis. The “content creator” which in this study refers to the individual or company who posted the Instagram post, was also recorded. Data categorisation was performed jointly by both data collectors (S.C. and G.M.) to minimise subjectivity, and any discrepancies were discussed with a third researcher (P.H.) until a consensus was reached. Data were compiled, and frequencies were reported using Microsoft Excel software.

### 2.3 Assessment of Quality

The DISCERN instrument [34] was used to evaluate the quality of the health information provided in the Instagram posts. This instrument has been used by previous research looking at the quality of social media content [24, 29, 32, 36]. The Instagram posts were evaluated in their entirety (before the splitting of some posts per dietary approach). Initially, 10% of the posts were rated by two researchers individually (S.C. and P.H.), and then compared, to ensure reliability. Any discrepancies in overall rating were discussed and a consensus was established. This reasoning was then used to guide the evaluation of the remaining posts, completed by one researcher (S.C.).

### 2.4 Ethical Considerations

This study did not require approval from an ethical committee because the data gathered was already publicly available and did not involve the recruitment of human participants. However, this research did follow the principles outlined in the British Psychological Society's Ethics Guidelines for Internet‐Mediated Research [37].

## 3 Results

In total, 1200 Instagram posts were identified; after excluding duplications and those that did not meet the inclusion criteria, 138 eligible Instagram posts were included for further analysis (Figure 1). The 12 most popular psoriasis‐related hashtags identified and subsequently searched are shown in Figure 1, of these, only one contained any reference to nutrition or diet which was ‘#psoriasisdiet’.

### 3.1 Dietary Approaches

**Figure 2:**
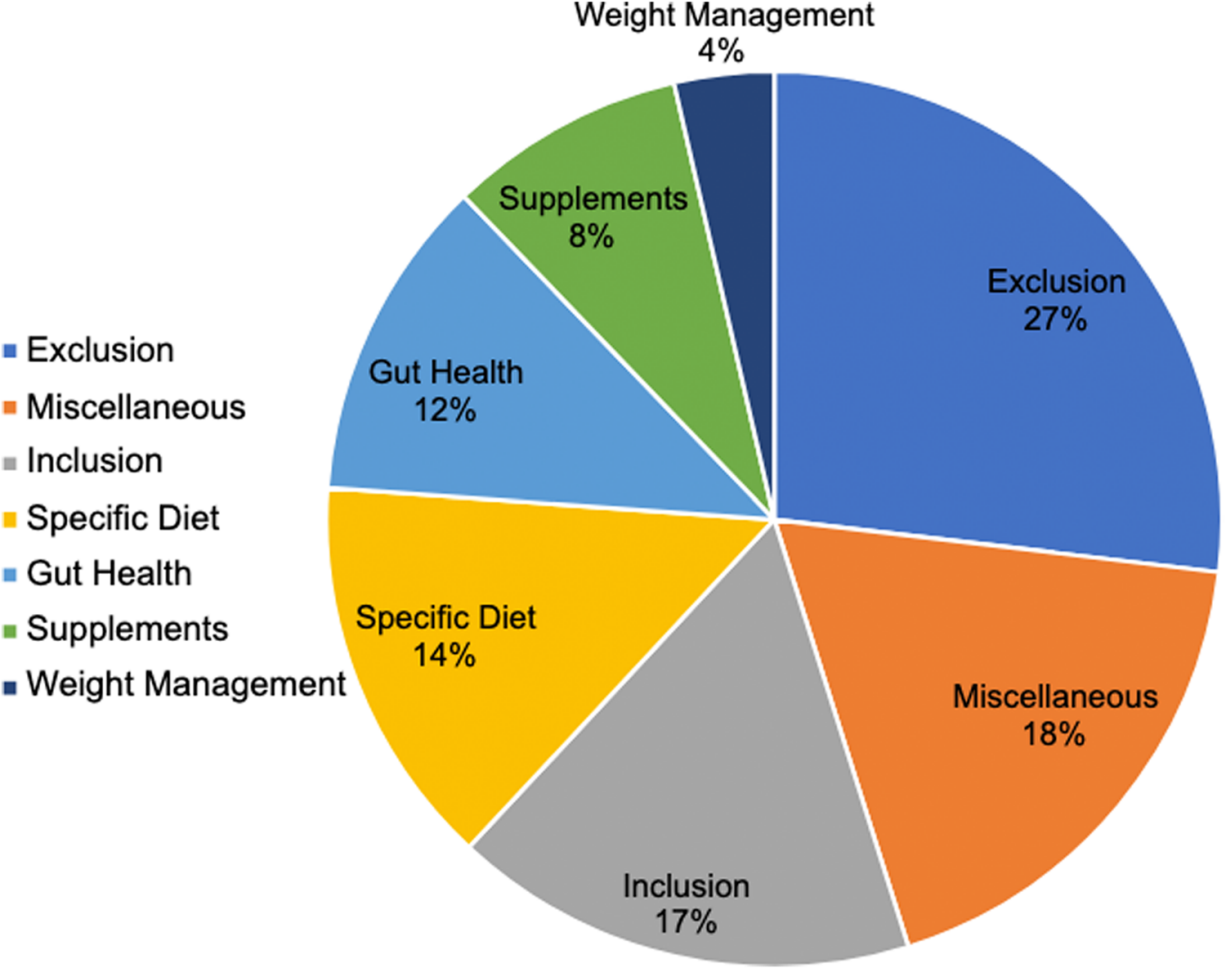
Shows the percentage (%) of Instagram posts identified under popular psoriasis hashtags recommending each type of dietary approach for the management of plaque psoriasis (*n* = 197).

Overall, 197 dietary approaches were identified in the 138 Instagram posts. The dietary approaches identified were grouped into seven categories depending on the type of dietary information given. The seven categories were as follows: (1) Inclusion, dietary approaches that suggested the addition or increased consumption of specific foods or food groups. (2) Exclusion: dietary approaches suggesting excluding a certain food or food group. (3) Specific diet: dietary approaches that suggest following a certain diet. (4) Weight loss: dietary approaches that suggest losing weight. (5) Supplements refer to dietary approaches that suggest taking specific vitamins, minerals or herbs. (6) Gut health refers to posts that suggested improving gut health using diet. (7) Miscellaneous refers to nonspecific dietary suggestions such as ‘detoxing’ and ‘what I eat in a day for my psoriasis’. The most common type of dietary approach recommended was exclusion, with 27% of posts recommending the exclusion of a specific dietary component. Miscellaneous dietary suggestions made up 18% of all posts, 17% recommended the inclusion of a dietary component, 14% recommended following a specific diet, 12% suggested improving gut health, 8% recommended taking a specific supplement and 4% recommended weight loss for the management of psoriasis (Figure 2).

Within the ‘exclusion’ category the most common recommendations were the exclusion of alcohol (28%) and dairy (11%). Within the ‘miscellaneous’ category detox diets and clean eating were the most common, with 42% of posts mentioning these and 36% of posts under this category providing anecdotal examples of diets or foods eaten and ‘what I eat in a day’ for psoriasis management. Within the posts under ‘inclusion’, the most common recommendations were the inclusion of protein (17%) and cruciferous vegetables (14%). Within the ‘Specific diet’ category, a gluten‐free diet was most suggested (19%), followed by autoimmune protocol (13%) and a vegan diet (13%). Within ‘gut health’, dietary information was often nonspecific with 73% of posts in this category mentioning that a ‘leaky gut’ can contribute to psoriasis or that improving ‘gut health’ or the ‘microbiome’ with diet can improve psoriasis. The remaining posts (27%) recommend the use of probiotics. In the ‘supplements’ category, the most common recommendation was to take vitamin D (32%). The posts within ‘weight loss’ all suggested losing weight. Each dietary approach category included another category, where dietary approaches were placed when they had not been identified frequently enough to be a separate subcategory. Within ‘exclusion’, dietary approaches which fell within the ‘other’ subcategory included saturated fatty acids, high histamine foods and citrus fruits. Within ‘inclusion’, the subcategories within ‘other’ included extra‐virgin olive oil, unsaturated fatty acids, turmeric, aloe vera, bone broth, oatmeal and antioxidants. The subcategories within ‘other’ in ‘specific diet’ included time‐restricted eating, low calorie, high alkaline diet, sugar‐free, whole‐food diet and anti‐inflammatory diet. Within ‘supplements’, dietary approaches under the ‘other’ subcategory included selenium, vitamin B‐12 and collagen. Further details of all the dietary recommendations made under each category can be seen in Table 1.

**Table 1:** The percentage of each specific dietary recommendation under each dietary approach category.

Dietary approach category	Specific type	% of each category
Exclusion (*n* = 53)		*%*
	Alcohol	28
	Dairy	11
	Processed foods	9
	Refined sugar	9
	Red meat	7
	Inflammatory foods	6
	Nightshades	6
	Egg yolk	4
	Caffeine	2
	Other (e.g., fatty acids, high histamine foods, citrus fruits)	18
Miscellaneous (*n* = 36)		***%***
	Detox or clean eating	42
	“What I eat in a day”/personal diet eaten to help psoriasis	36
	Promotions: e.g., diet and psoriasis plans, supplement mix	19
	Does not think that diet helps psoriasis	3
Inclusion (*n* = 33)		***%***
	Protein	17
	Cruciferous vegetables	14
	Fruits	11
	Herbal tea	11
	Nuts and seeds	9
	Anti‐inflammatory foods	9
	Other (e.g., extra‐virgin olive oil, unsaturated fatty acids, bone broth)	29
Specific diet (*n* = 28)		***%***
	Gluten‐free diet	19
	Autoimmune protocol	13
	Vegan	13
	Nonspecific mention of a “Healthy diet”	10
	Low carbohydrate	6
	Carnivore	6
	Nonspecific mention of an “Elimination diet”	6
	Other (e.g., time‐restricted eating, low calorie, high alkaline diet)	26
Gut health (*n* = 23)		***%***
	Nonspecific mention of “gut health”	73
	Probiotics	27
Supplements (*n* = 17)		***%***
	Vitamin D	32
	Naturopathic herbs	16
	Omega 3	16
	Zinc	16
	Other (e.g., selenium, vitamin B‐12 and collagen)	21
Weight loss (*n* = 7)		***%***
	Weight loss	100

### 3.2 Content Creators

The type of content creator for each post (*n* = 138) included was collected and analysed. The following seven categories were established: (1) ‘qualified nutrition professional’; (2) ‘nutrition background’; (3) ‘lived experience’; (4) ‘complementary and alternative medicine’ (CAM); (5) ‘healthcare professional’ (HCP); (6) ‘health and beauty company’ and (7) ‘other’. To classify as a ‘qualified nutrition professional’, the content creator must have clearly indicated on their profile a degree level nutrition or dietetics qualification which is regulated by an official body, for example, ‘dietitian’ or ‘RD’, ‘registered nutritionist’ or ‘ANutr/RNutr’ or something specific to suggest the completion of an accredited degree or registration with a recognised health professional body [29]. Whereas for ‘nutrition background’, the content creator does not clearly indicate whether they have an accredited qualification or if they are registered with a recognised health professional body, for example, ‘nutritionist’. The ‘healthcare professional’ category included content creators that clearly indicated that they were a registered HCP, but non‐diet or nutrition related for example ‘nurse’ or ‘dermatologist’. This distinction was made to separate them from registered nutrition and dietetics professionals, who have their own category. For ‘lived experience’, the content creator must have clearly indicated on their profile or the Instagram post that they live with psoriasis. Regarding the ‘other’ category, this included content creators with no indication of a specific category or with a vague title such as ‘health coach’.

**Figure 3:**
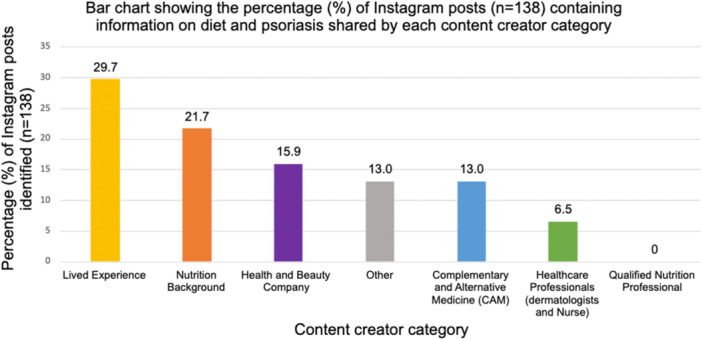
Bar chart of the percentage (%) of posts created or shared by each content creator category.

The results showed that the most frequent content creator was ‘lived experience’ with 29.7% of the identified Instagram posts created or shared by an individual with psoriasis, followed by ‘nutrition background’ (21.7%). The least common content creator was ‘healthcare professional’ with only 6.5% of posts created or shared by this group. Interestingly, no content creators fell within the ‘qualified nutrition professional’ category (Figure 3).

**Figure 4:**
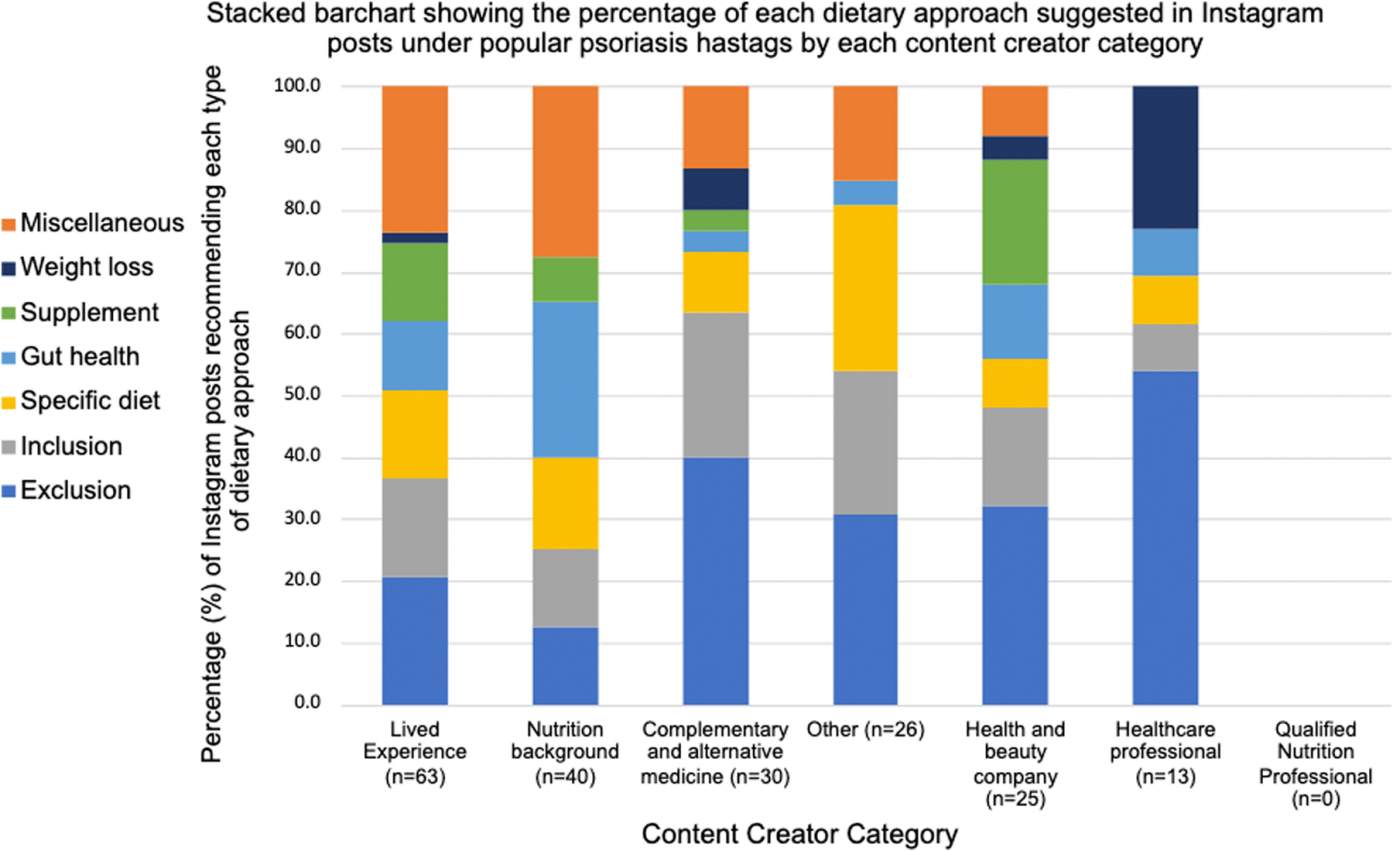
Stacked bar chart of the percentage of dietary approach type created or shared by each content creator.

The dietary approaches and the type of content creator categories were analysed to ascertain the type of dietary approaches shared by the different types of content creators. The most common dietary approaches recommended by content creators with ‘lived experience’ was ‘miscellaneous’ (23.8%) and ‘exclusion’ (20.6%). For those in the ‘nutrition background’ category, ‘miscellaneous’ was also the most recommended dietary approach, with 27.5% of all posts created or shared by this type of content creator falling under this category, alongside gut health (25%). Dietary approaches that were exclusionary were the most common type of dietary approach recommended by those in the CAM category (40%). Regarding the health and beauty companies, exclusion and supplements were the most shared type of dietary approach recommended to help manage psoriasis, with 32% and 20% of their posts containing this type of information, respectively. Within the HCP category, exclusion (53.8%) and weight loss (23.1%) were the most common dietary approaches recommended. No HCPs provided information on supplements or miscellaneous dietary approaches. See Figure 4 for a full overview of the type of dietary approach shared by each content creator category.

### 3.3 DISCERN Instrument Rating

Overall, the quality of the 138 Instagram posts was poor, with 99% (*n* = *136*) of posts rated as ‘low’ quality using the DISCERN instrument. Just 1% (*n* = *2*) of the posts rated as *‘*moderate’ quality (Table 2.). The ‘moderate’ quality posts were posted by dermatologists within the ‘healthcare professional’ category. No posts scored highly according to the DISCERN instrument. The user guide for the DISCERN rating given for each question and overall score for each Instagram post are detailed in Appendix 1 [34].

**Table 2:** Percentage of DISCERN scores given across the content creator categories for Instagram posts about diet and psoriasis, using the DISCERN instrument to assess their quality (*n* = 138).

DISCERN score	Nutrition background	Lived experience	Complementary and alternative medicine	Healthcare professional	Health and beauty company	Other	Total
Low ‘poor’ quality	100%	100%	100%	78%	100%	100%	99%
Moderate ‘fair’ quality	0%	0%	0%	22%	0%	0%	1%
High ‘good’ quality	0%	0%	0%	0%	0%	0%	0%

*Note:* Red = Low ‘poor’ quality rating of posts using the DISCERN score. Yellow = Moderate ‘fair’ quality rating of posts using the DISCERN score. Green = High ‘good’ quality rating of posts using the DISCERN score.

## 4 Discussion

To the best of our knowledge this is the first study that has explored the content and quality of dietary information being posted on Instagram regarding psoriasis management. The findings suggest that most of the dietary information for psoriasis on Instagram is of poor quality, shared by non‐HCPs and commonly suggests restrictive and unsubstantiated dietary advice.

Concerningly, the DISCERN instrument exposed extensive shortcomings in the quality of most Instagram posts about diet and psoriasis, with 99% found to be poor quality. This is greater than previous research on the quality of psoriasis‐related Instagram posts [24], suggesting that nutrition‐specific information on Instagram is more prone to being of worse quality than general psoriasis health information. The poor ratings in this study relate not only to the lack of scientific evidence base, but its combination with biased language, lack of uncertainty and risk acknowledgement, and lack of signposts to possible alternatives or additional support. These findings corroborate other studies which have drawn similar conclusions concerning health information across social media platforms, spanning conditions more than just psoriasis [24, 29, 33, 36, 38].

Overall, ‘exclusion’ was the most frequent type of dietary approach recommended for psoriasis management in the identified Instagram posts. The most common exclusions were alcohol and dairy. Alcohol has been implicated in the development and exacerbation of psoriasis severity [39, 40]. The evidence to support dairy's role in psoriasis is lacking, however, its elimination could result in the removal of a major source of protein and important micronutrients such as iodine, zinc and calcium [41, 42]. A blanket recommendation to remove food groups may not only be ineffective as a psoriasis management strategy but could result in nutritional deficiencies, especially if followed without HCP guidance [8, 16, 17, 42]. Among the dietary approaches mentioned in the posts, weight loss was the least commonly recommended, despite having the most robust evidence for psoriasis management in individuals living with overweight or obesity [8, 9].

A concerning finding from this study was that no content creators who shared dietary information were classified as a ‘qualified nutrition professional’. This lack of online HCP presence is another unanimous finding across other specialities [24, 29, 30, 43]. The information shared by a degree‐level qualified registered and/or accredited nutrition professional, such as a registered dietitian, is held against professional and legal standards by regulatory bodies such as the ‘health and care professions council’ (HCPC) [25, 43, 44, 45]. This is crucial because it ensures that the information is safe and trustworthy. This contrasts with nonregulated nutrition professionals, or those with a ‘nutrition background’, whose information does not reliably ensure the same [46]. This is further supported by research findings that a registered dietitian is more likely to give safe and healthy dietary advice when compared to a non‐registered dietitian [25]. Worryingly, research indicates that the general public is not familiar with the differences in these nutrition titles [47], which leaves PLwP at risk of following unregulated advice [48].

Those with ‘lived experience’ were the most common content creators, with 33% of posts shared by them. This is greater than those with lived experience of psoriasis posting on Twitter (20%) [28] and YouTube (15.5%) [30]. This suggests that PLwP may turn to Instagram more than other social media platforms, possibly because Instagram is an especially visual platform with relative ease of use [49]. The content produced by those with lived experience of psoriasis is often more personal, anecdotal and relatable than other posts [49, 50]. These anecdotes can be even more compelling to PLwP than clinical trial evidence [27, 51, 52]. ‘Lived experience’ content creators frequently recommended making dietary exclusions. These suggest that PLwP are already practicing restrictive dietary practices or may be likely to trial them as they are recommended by other PLwP. This is an important insight that HCPs can use to provide better support to PLwP, tailoring dietary counselling towards safe dietary practices and limiting unnecessary dietary exclusions. Whilst these posts may not be a source of robust science, the content posted by PLwP provides psychosocial support for those living with psoriasis [28, 49] and has been shown to reduce psoriasis stigma on Instagram [53], so their utility should not be completely disregarded.

Content creators with a ‘nutrition background’ were the second most common type of content creator sharing diet‐related posts under popular psoriasis hashtags. They often promoted restrictive ‘exclusion’ dietary modifications and ‘miscellaneous’ dietary approaches. These included recommendations for detoxes and cleanses, diet plan promotions and ‘what I eat in a day’ type posts. These types of posts have previously been shown to result in the recirculation of misinformation [54]. Health and beauty companies most frequently suggested taking supplements under psoriasis hashtags, indicating that PLwP may be targeted by companies promoting products. Consumers should be critical of nutrition information from companies. However, stronger regulation is needed to protect Instagram users from misinformation driven by commercial interests.

Only 6.5% of posts on diet and psoriasis were shared by HCPs. Despite this, they were responsible for the Instagram posts that rated as ‘moderate’ quality using the DISCERN instrument. The moderate scoring was attributed to making explicitly clear the source and date of information used to create the Instagram posts, using unbiased language and having adequately clear aims. However, these posts still fall short of being considered a good‐quality source of health information. A recent study exploring the dietary knowledge of dermatology professionals found although the majority reported being asked dietary questions by patients, 73.1% did not feel confident in their ability to answer them [55]. This emphasises that PLwP are best supported when receiving dietary advice from a qualified nutrition professional. Furthermore, of the 12 hashtags under which these posts were identified, only one contained any mention of diet. This suggests that PLwP who use Instagram may be subject to dietary information, which this study highlights is often unsubstantiated and poor quality, even if they do not explicitly search for it. All of which further emphasise the need for dietary support to be made available to this population. This is particularly important considering the associated co‐morbidities, and lack of qualified nutrition professionals providing evidence‐based nutrition information on social media.

This study has strength in the sample size, allowing a comprehensive expansion of previous research looking into the quality of psoriasis information on Instagram [24]. The novel findings provide insights into the dietary misinformation that PLwP may be subject to. The limitations included the potential for mis‐categorising content creators into ‘nutrition background’ instead of ‘qualified nutrition professional’ due to challenges created by differences in nutrition training, professional registration and accreditation, and the different professional titles used in nutrition [18, 46, 56]. It was not possible to obtain a formal psoriasis diagnosis for ‘lived experience’ content creators. Data was only collected from public accounts, and not private accounts, therefore findings may not be generalisable. Measures were taken to avoid bias from Instagram algorithms; however, search results may have been affected outside of the researcher's control. Seeking health information online is a relatively novel concept and there is a lack of validated tools available specifically designed for evaluating the quality of social media posts; the DISCERN tool [34] was originally intended to evaluate written health information rather than social media. Future research should explore psoriasis‐related dietary information on other social media platforms and take into consideration the popularity of each post.

## 5 Conclusion

The findings of this study highlight that Instagram is a source of psoriasis‐related dietary information, with most posts found to be of poor quality and unsubstantiated. Concerningly, none of the nutrition information came from a qualified nutrition professional. Rather, most commonly from creators with lived experience or a nutrition background. This emphasises the need for evidence‐based guidance to help PLwP navigate the plethora of dietary advice and misinformation they may encounter. Alongside ensuring that HCPs are equipped to offer dietary support and combat misinformation, to enhance patient‐centred care for PLwP. Additionally, further exploration into how PLwP perceive or use the dietary information found on social media, and how HCPs can support PLwP will increase understanding of ways to provide dietary support to PLwP, amid the lack of evidence‐based guidelines.

## Author Contributions

Sabrina Cowan, Rosalind Fallaize and Poppy Hawkins were responsible for research conceptualisation and study design. Sabrina Cowan and Poppy Hawkins were responsible for the methodology. Sabrina Cowan and Ghislaine Marks performed the data collection. Sabrina Cowan was the principal author responsible for the original draft preparation. Poppy Hawkins and Rosalind Fallaize contributed to reviewing the manuscript. All authors have reviewed and agreed to the published version of the manuscript.

## Conflicts of Interest

The authors declare no conflicts of interest.

### 1 Peer Review

The peer review history for this article is available at https://www.webofscience.com/api/gateway/wos/peer-review/10.1111/jhn.70034.

## Supporting information

Supporting information.

## Data Availability

The data that support the findings of this study are available from the corresponding author upon reasonable request.
