# Supplementary material for: Diet and Psoriasis‐Related Information on Instagram: A Quality and Content Analysis of Posts Under Popular Psoriasis Hashtags
Source: J Hum Nutr Diet. 2025 Mar 4;38(2):e70034. doi: 10.1111/jhn.70034 (PMC11880412; doi:10.1111/jhn.70034)
Supplement: Supplementary file 1 — Supporting information. [file JHN-38-0-s001.docx]

| **Appendix 1.** Researcher-made user guide for the DISCERN instrument ratings specific to research topic. | | | | |
| --- | --- | --- | --- | --- |
| **No.** | **Summary** | **Explanation** | **Score** | **Notes** |
| *Evaluating the reliability…* | | | | |
| 1 | Have explicit aims | *Are the aims clear?* | 1 = No, there is no clear indication about what the post is about, what is going to be covered nor who it is aimed towards  2-4 = There is some reference to at least one of the following: what the post is about, what it will cover and who may find it useful  5 = Yes, it is explicitly clear what the post is about, what it is meant to cover and who might find it useful | **If the answer to Question 1 is ‘No’, go directly to Question 3** |
| 2 | Achieve its aims | *Does it achieve its aims?* | 1 = No, it does not achieve its aims  2-4 = it partially achieves the aims set out above  5 = Yes, all of the aims described above are met | Consider whether the publication provides the information it aimed to as outlined in Question 1. |
| 3 | Relevance | *Is it relevant?*  *Does the Instagram post provide information about dietary changes in relation to psoriasis?*  A good quality post about treatment choices should be relevant to the lifestyle and circumstances of someone with psoriasis | 1 = No, the information is not relevant to diet and psoriasis  2-4 = The information is relevant but makes unrealistic recommendations, contains assumptions or language that is inappropriate  5 = Yes, the post is about dietary approaches in relation to psoriasis | Consider whether:   - the publication addresses the questions that readers might ask - recommendations and suggestions concerning treatment choices are realistic or appropriate. |
| 4 | Explicit Sources of information | *Is it clear what sources of information were used to compile the publication (other than the author or producer)?*  A good quality post would make clear where the evidence about psoriasis and diet has come from. | 1 = No, statements of facts are not accompanied by a reference, and there is no reference list at the end.  2-4 = An evidence source may be alluded to within the post, but there is no explicit mention of the article or author. An author’s name may be mentioned, but the relevant study is not named. There may be a referenced state of fact with no reference list at the end, or vice versa.  5 = Yes, each statement of fact is accompanied by a reference AND the post has a full reference list at the end | **A high rating on this question does not mean that the information is accurate or of good scientific quality.**  It tells you it meets our criterion of the sources of evidence being explicit. |
| 5 | Explicit date of information | *Is it clear when the information used or reported in the publication was produced?*  a good quality publication will make the date of the information about treatment choices explicit  Both the date of publication (ie the date of posting to Instagram) and the date for all acknowledged sources of information should be provided. | 1 = No, no dates have been given.  2-4 = only the date of the publication itself (ie the date which the Instagram post was published) is clear, or dates for some but not all acknowledged sources have been given. There may be mention of the words ‘recent’, ‘new’, ‘old’ etc with regards to the date  5 = Yes, dates for the main sources of information used to compile the publication are provided | DISCERN cannot be used to assess how ‘up-to-date’ the information is, as the rate of change will vary with each medical condition and treatment.  The publication cannot be older than the sources of evidence, whereas the sources of evidence can be much older than the publication. Therefore, in order to fulfil the quality criterion for Question 5, the dates for the sources of evidence identified in Question 4 must be clear. A publication that has rated 1 on Question 4 cannot rate high on Question 5. |
| 6 | Balanced and unbiased | *Is it balanced and unbiased?*  *Does the Instagram post provide unbiased opinions on dietary approaches in relation to psoriasis?*  A good quality publication will provide fair and impartial information, presented in a way that enables you to choose what is in your best interests.  A publication should be honest and informative. It should not influence you by ‘promoting’ particular treatment choices or by using ‘shock tactics’. | 1 = No, the information is completely unbalanced or biased (ie there is no acknowledgement of other options or they are unfairly dismissed, there are false promises made, language is biased and scaremongering or appeals to emotion, the author claims the treatment is suitable for everyone)  2-4 = Some aspects of the information are unbalanced or biased (there may be some of the above, but there may also be some more fair/unbiased information provided)  5 = Yes, there are a range of sources of evidence explicitly given, the information is impartial and fair, | You should judge the information on its own merits and you should not be influenced by what you know about the author or producer.  Publications describing one particular treatment choice can be acceptable if the author has made this clear (Question 1) and has acknowledged that other treatment choices may be available (Question 14). In all cases, the information about the treatment choice or choices should be drawn from a range of research and experience. **You should not give a high rating to a publication that relies solely on a single source of evidence or has not revealed any sources** (Question 4).  Look for:   - clear indication of whether the publication is written from a personal or objective point of view - evidence that a range of sources of information was used to compile the publication, e.g. more than one research study or expert   Be wary if:   - the publication relies primarily on evidence from single cases (which may not be typical of people with this condition or of responses to a particular treatment) - the information is presented in a sensational, emotive or alarmist way. |
| 7 | Additional support | *Does it provide details of additional sources of support and information?*  Details of other sources of support and information about treatment choices are important, as the publication may not provide you with all the information you need and you should be able to trace further information easily | 1 = No, additional sources of support and information are not given  2-4 = the publication provides details of an additional source or sources, but the details are incomplete or consist only of local branches of the same organisation  5 = Yes, there are detailed signposts to other sources of information for psoriasis and diet to be easily found, such as reading materials or organisations | A reference list or bibliography can also be considered further reading. The details provided should enable you to find the additional sources easily, e.g. name, address and telephone number in the case of an organisation, and author, title, publisher or producer in the case of reading material |
| 8 | Areas of uncertainty | *Does it refer to areas of uncertainty?*  *Does the post describe areas of uncertainty with regards to the suggested dietary approach?*  A good quality publication will include a reference to ‘grey’ areas where there is uncertainty about the most effective treatment, such as no evidence about effective treatment choices exists, the existing evidence is contradictory, or there is uncertainty as to who is most likely to benefit or be at risk from the treatment choice. | 1 = No, no uncertainty about treatment choices is mentioned.  2-4 = uncertainty is mentioned but the information is unclear or incomplete.  5 = Yes, the publication includes a clear reference to any uncertainty regarding treatment choices: this may be linked to each treatment choice or may be covered in a more general discussion or summary of the choices mentioned in the publication. | A good quality publication will highlight the fact that the choice of the most suitable treatment may not be clear-cut and that it may not be possible to predict the most likely outcome for you.  Be wary if the publication implies that a treatment choice affects everyone in the same way, e.g. 100% success rate with a particular treatment. |
| *Evaluating the treatment choices…*  NB – apart from question 14, the questions are concerned with the treatment choice or choices described in the publication, and not with all the possible treatment choices.  NB - Questions 9 to 11 are concerned with the ‘active’ treatments described in the publication and can include self-care. ‘No treatment’ options are dealt with separately in Question 12 | | | | |
| 9 | Explaining the treatment | *Does it describe what the dietary approach entails and how it will affect plaque psoriasis symptoms?*  A good quality publication will include information about how a treatment acts on the body and in what way it ‘treats’ or affects the condition or its symptoms. | 1 = No, none of the descriptions about the dietary approaches include details of how treatment works.  2-4 =  5 = Yes, there is clear description of how each dietary approach mentioned works to affect psoriasis including mention about the timescale |  |
| 10 | Benefits of treatment | *Does it describe the benefits of each dietary approach?*  The question is only concerned with the treatment or treatments described in the publication, and simply requires you to rate whether any benefit is mentioned for each of the treatments described | 1 = No, no benefits are described for any of the dietary approaches  2-4 = a benefit is described for some but not all of the dietary approaches.  5 = Yes, a benefit is described for each dietary approach mentioned | The question is not concerned with the size of the benefit or who is most likely to benefit. The question cannot be used to assess whether all of the benefits associated with each treatment have been described as this would involve checking against other sources  Benefits can include controlling or getting rid of symptoms, preventing recurrence of the condition and eliminating the condition, both short-term and long-term. |
| 11 | Risks of treatment | *Does it describe the risks of each dietary approach?*  The question is only concerned with the dietary approaches described in the publication, and simply requires you to rate whether any risk is mentioned for each of the treatments described.  The question is not concerned with the size of the risk or who is most likely to be at risk. | 1 = No, no risks are described for any of the dietary approaches  2-4 = a risk is described for some but not all the dietary approaches  5 = Yes, a risk is described for each dietary approach | The question cannot be used to assess whether all of the risks associated with each dietary approach have been described as this would involve checking against other sources.  Risks can include side-effects, complications and adverse reactions to treatment, both short-term and long-term. |
| 12 | Consequence of no treatment | *Does it describe what would happen if no treatment is used?*  A good quality publication will include a description of what would happen if the condition is left ‘untreated’. | 1 = No, there are no consequences of no treatment discussed    2-4 = the post alludes to there being consequences of no treatment  5 = Yes, there is a clear description of a risk or a benefit associated with any no treatment option. | The question simply requires you to rate whether any outcome associated with not using treatment is mentioned. The question is not concerned with the size of the risks or benefits or who is most likely to be at risk or to benefit from no treatment options. It is not possible to assess whether all the risks or benefits of each no treatment option have been described as this would involve checking against other sources.  Look for a description of the risks and benefits of postponing treatment, of watchful waiting (i.e. monitoring how the condition progresses without treatment) or of permanently forgoing treatment. |
| 13 | Quality of life | *Does it describe how the dietary approach will affect overall quality of life?*  A good quality publication will include a description of the broader aspects of treatment choices – not just risks and benefits, but the overall impact of a treatment choice or choices on day-to-day living. It may involve major changes in lifestyle or circumstances or have important effects on those close to you that you need to be aware of and consider before making a decision. | 1 = No, there is no reference to overall quality of life in relation to dietary changes  2-4 = the publication includes a reference to overall quality of life in relation to dietary changes, but the information is unclear or incomplete.  5 = Yes, the publication includes a clear reference to overall quality of life in relation to *any* of the dietary approaches mentioned. | Look for:   - description of the effects of the treatment choices on day-to-day activity - description of the effects of the treatment choices on relationships with family, friends and carers. |
| 14 | Possible alternatives | *Is it clear that there may be more than one possible dietary approach?*  The publication should indicate that the dietary approaches described may be suited to some people more than others, and that there is nearly always a choice of treatment, even if a full account of alternatives has not been presented in the publication. | 1 = No, the publication does not give any indication that there may be a *choice* about dietary approaches  2-4 = the publication indicates that there may be more than one possible choice, but the information is unclear or incomplete  5 = Yes, the publication makes it very clear that there may be more than one possible dietary change | Look for:   - a description of who is most likely to benefit from each treatment choice mentioned, and under what circumstances - suggestions of alternatives to consider or investigate further (including choices not fully described in the publication) before deciding whether to select or reject a particular treatment choice. |
| 15 | Shared decision making | *Does it provide support for shared decision-making?*  A good quality publication will raise issues for you to discuss with all those involved in your care about the best treatment choice for you. The information about a treatment choice or choices provided by the publication should enable you to prepare for a consultation with a health professional or to talk through issues that might affect your family, friends or carers regarding your treatment choices. | 1 = No, the publication provides no support for shared decision making  2-4 = the publication provides some support for shared decision making.  5 = Yes, the publication provides very good support for shared decision making | These issues should be made clear throughout the publication, rather than merely being queries arising from its deficiencies and gaps.  How high you rate the publication will depend on your judgement of how much it will support you in sharing decisions about treatment choices.  Look for suggestions of things to discuss with family, friends, doctors or other health professionals concerning treatment choices.  ‘eg should you fall pregnant, you should talk to your doctor about how to support your pregnancy with this dietary approach’’  Eg ‘you may wish to ask your healthcare provider for advice on which supplements to take with this dietary approach’  Eg ‘talk with your friends and family about how they can support you with this dietary approach during social occasions’ |
| *Regarding the overall quality rating…* | | | |  |
| 16 | Overall quality rating | Based on the answers to all of the above questions, rate the overall quality of the publication as a source of information about treatment choices | Low (1) – the publication rated low (2 or below) on the majority of questions. A low overall quality rating indicates the publication is ‘poor’ quality – it has serious shortcomings and is not a useful or appropriate source of information about treatment choices. It is unlikely to be of any benefit and should not be used.  Moderate (3) – the publication rated high and low on a similar number of questions, or the majority of questions rated in the mid-ranges (3). A moderate overall quality rating indicates the publication is ‘fair’ quality – it is a useful source of information about treatment choices but has some limitations. Additional information or support would definitely be needed.  High (5) – the publication rated high (4 or above) on the majority of questions. A high overall quality rating indicates the publication is ‘good’ quality – it is a useful and appropriate source of information about treatment choices. |  |
| *Note.* Adapted from: Charnock, D., Shepperd, S., Needham, G. and Gann, R. (1999) DISCERN: an instrument for judging the quality of written consumer health information on treatment choices, *Journal of Epidemiology and Community Health*, 53(2), pp. 105–111. | | | | |
